# Screening for alcohol-related liver damage in the community: findings from the PrevAIL (Preventing Alcohol Harm in Liverpool) Study

**DOI:** 10.1186/1940-0640-8-S1-A18

**Published:** 2013-09-04

**Authors:** Penny A Cook, Michela Morleo, David Billington, Mark Gabbay, Nick Sheron, Ian T Gilmore, Mark A Bellis

**Affiliations:** 1 School of Health Sciences, University of Salford, Greater Manchester, UK; 2 Centre for Public Health, Liverpool John Moores University, Liverpool, UK; 3 School of Biomolecular Sciences, Liverpool John Moores University, Liverpool, UK; 4 Health Services Research, University of Liverpool, Liverpool, UK; 5 Medical School, University of Southampton, Southampton, UK; 6 Royal Liverpool University Hospital, Liverpool, UK

## 

Progression of alcohol-related liver fibrosis stops when drinking stops, but the diagnosis is usually missed because the process of fibrosis is symptom free and missed by the usual liver function tests. Non-invasive tests to detect fibrosis and cirrhosis are available, but not currently used in primary care. We aimed to: identify optimal ways of engaging communities with liver disease screening; to inform a future trial to augment brief interventions with a liver risk score; and to estimate the prevalence of liver disease. Participants, aged 36-55y, registered with general practice (GP) or working in Merseyside, UK, were contacted by post (GP) or through workplaces. Risky drinkers (previous week drinking >112g females/168g males) were invited for a liver screen. Blood samples were tested for fibrosis markers (hyaluronic acid and procollagen type III N-terminal peptide) and categorised using the Simple Traffic Light (STL) algorithm. Of 6439 GP registrants, 539 (8%) returned the alcohol consumption questionnaire; 152 were risky drinkers and were invited for liver screening, and 27 attended. Screening in the 13 participating workplaces (out of 37 approached) was attended by 2-6% of the eligible workforce (n=363). Of 142 risky drinkers, most (91%) accepted the liver screening test. In total, seven samples were graded ‘red’, yielding a prevalence of 4.6% (95%CI 2.02—9.14%) of probable liver disease and further 26.3% (20.0—33.7%; 41 samples) scored ‘amber’ (moderate risk). Detecting and supporting cases in the community could avert deaths and save costs, and this work informs development of a trial to determine whether feedback of liver disease risk scores is more effective than brief intervention alone. We conclude that workplaces are optimum sites, because screening takes place at a time and location that was convenient for participants; however alternative methods will be required to access those who do not work, whose risk may be higher.

